# 3D printed patient-specific prostate cancer models to guide nerve-sparing robot-assisted radical prostatectomy: a systematic review

**DOI:** 10.1007/s11701-022-01401-0

**Published:** 2022-03-29

**Authors:** Jasamine Coles-Black, Sean Ong, Jiasian Teh, Paul Kearns, Joseph Ischia, Damien Bolton, Nathan Lawrentschuk

**Affiliations:** 1 Department of Surgery, Austin Health, University of Melbourne, 145 Studley Road, Heidelberg, Melbourne, VIC 3084 Australia; 2 Young Urology Researchers Organisation (YURO), Melbourne, Australia; 3 EJ Whitten Prostate Cancer Research Centre, Epworth Healthcare, Melbourne, Australia; 4 Division of Cancer Surgery, Peter MacCallum Cancer Centre, Melbourne, Australia; 5 Olivia Newton-John Cancer Research Institute, Melbourne, Australia; 6 Department of Surgery, The Royal Melbourne Hospital, University of Melbourne, Melbourne, Australia

**Keywords:** Robotic surgery, 3D printing, Prostate cancer, RARP, NS-RARP

## Abstract

Precise knowledge of each patient’s index cancer and surrounding anatomy is required for nerve-sparing robot-assisted radical prostatectomy (NS-RARP). Complementary to this, 3D printing has proven its utility in improving the visualisation of complex anatomy. This is the first systematic review to critically assess the potential of 3D printed patient-specific prostate cancer models in improving visualisation and the practice of NS-RARP. A literature search of PubMed and OVID Medline databases was performed using the terms “3D Printing”, “Robot Assisted Radical Prostatectomy” and related index terms as per the Preferred Reporting Items for Systematic Reviews and Meta-Analyses (PRISMA) guidelines. Eight articles were included; six were identified via database searches, to which a further two articles were located via a snowballing approach. Eight papers were identified for review. There were five prospective single centre studies, one case series, one technical report and one letter to the editor. Of these articles, five publications (62.5%) reported on the utility of 3D printed models for NS-RARP planning. Two publications (25%) utilised 3D printed prostate models for simulation and training, and two publications (25%) used the models for patient engagement. Despite the nascency of the field, 3D printed models are emerging in the uro-oncological literature as a useful tool in visualising complex anatomy. This has proven useful in NS-RARP for preoperative planning, simulation and patient engagement. However, best practice guidelines, the future regulatory landscape, and health economic considerations need to be addressed before this synergy of new technologies is ready for the mainstream.

**Supplementary Information:**

The online version contains supplementary material available at 10.1007/s11701-022-01401-0.

## Introduction

The applications of three-dimensional (3D) printing in Urology have been advancing rapidly in recent years. Better known in the engineering fields as additive manufacturing, the technology allows for objects to be built up ‘from nothing’ in a layered fashion. 3D printing allows for the rapid and accessible creation of complex geometries that were previously impossible to achieve with traditional manufacturing techniques [1].

As the technology has entered the mainstream consciousness, Urologists have capitalised on its strengths to 3D print anatomical models within the hospital environment [2, 3]. The technology allows for the creation of patient-specific models for visual and tactile interrogation, positioning it as a natural adjunct for complex cases where adequate visualisation of each patient’s unique anatomy is paramount [4].

Due to the enhanced visualisation offered by the technology, 3D printing in Urology has seen utility mainly in preoperative planning [5–9], simulation and training [10, 11], and patient education [8, 12]. However, to date health technology assessment of 3D printing in Urology is limited, with most studies comprising low-level evidence such as case series or technical reports. As with any new technology, lack of exposure and expertise currently limits the application of 3D printing in the broader healthcare ecosystem.

In contrast, after an initial lag in implementation, robot-assisted surgery has been adopted into widespread use. Robot-assisted radical prostatectomy (RARP) is a mainstream procedure, associated with lower perioperative morbidity and positive margin rates when compared to laparoscopic surgery, and higher postoperative continence rates when compared to a retropubic approach [13].

**Fig. 1:**
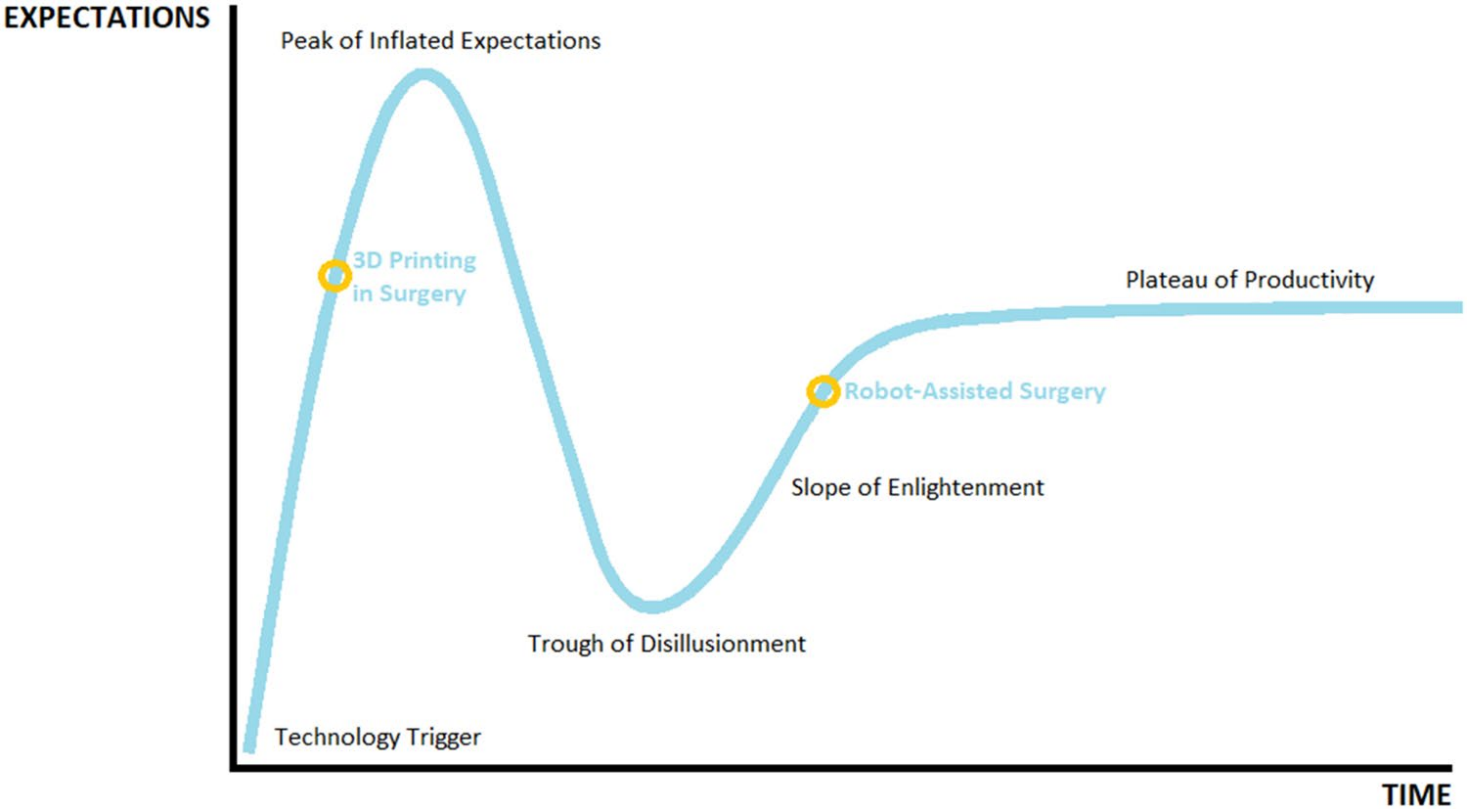
The current progression of 3D printing and robotic surgery on Gartner’s hype cycle

The ethical implementation of new technologies in surgery has been studied through the IDEAL guidelines [14], while the implementation of innovations into mainstream medicine can be described via their progress through the Gartner hype cycle [15]. 3D printing and robotic surgery currently occupy different stages on Gartner’s hype phase of technology. Due to its broad acceptance, uro-oncologic robotic surgery is currently ascending the ‘Slope of Enlightenment’. In comparison, despite widespread use in the field of engineering, 3D printing in surgery is a more recent application of the technology and has entered a stage of exponential growth [2] after an initial ‘Technology Trigger’. Contemporary medical innovation does not occur in a vacuum, with the intersection of new technologies becoming increasingly common (see Fig. 1).

Precise three-dimensional knowledge of each patient’s neurovascular anatomy is required for nerve-sparing robot-assisted radical prostatectomy (NS-RARP) to be feasible [16, 17]. When managing the dichotomy between postoperative functional outcomes and negative surgical margins, detailed knowledge of the anatomy of the index cancer and its relationship to the neurovascular bundle guides surgical decision-making. In many cases, preservation of the neurovascular bundle can be intended preoperatively; however, subjective intraoperative decision-making has been found to be the most accurate indicator of the necessity to sacrifice the neurovascular bundle [17] or undertake a wider resection with partial nerve sparing.

As such, there is a clear need for improved visualisation of the intimate relationship between structures when deciding on an approach during NS-RARP, which may be aided by 3D printing. This is the first systematic review to critically assess the potential of 3D printed patient-specific prostate cancer models in improving visualisation and the practice of NS-RARP.

## Methods

Due to the nascency of the field, we adopted a broad search strategy to capture all relevant articles. The search strategy was undertaken following the Preferred Reporting Items for Systematic Reviews and Meta-analyses (PRISMA) guideline [18]. This review was registered on the PROSPERO register (CRD42021276106).

### Search strategy

A literature search of PubMed and OVID Medline databases was performed using the search query (“3D Printing” OR “Three-dimensional printing”) AND (“Robot Assisted Radical Prostatectomy” OR “Robotic Assisted Laparoscopic Prostatectomy”). No filters were placed on the search which included articles in all languages and a timeline from inception to 13th August 2021. An updated search was also performed on 1st January 2022. Additionally, reference lists of all screened articles were interrogated.

### Inclusion and exclusion criteria

All articles published in English before the search date (1st January 2022) that reported data of interest were included in the review. Systematic and narrative reviews and conference abstracts that were later published were excluded. Abstracts were individually screened by all authors for eligibility and any disagreements were resolved by discussion until a consensus was reached. No automated tools were used in this process. Articles deemed eligible for this review were categorised into surgical planning, simulation, and patient engagement.

### Data extraction

Eight articles met inclusion criteria for this review. Six were identified via database searches, to which a further two articles were “snowballed” via a thorough appraisal of the reference list of known publications. The following data were extracted from all eligible articles; authors, study type, study size, software used, 3D printing technique, anatomy demonstrated and cost. Given the heterogeneity of the studies, a meta-analysis was not performed. Specific data relating to each study’s endpoints and aims were extracted for the review discussion.

### Risk of bias assessment

**Fig. 2:**
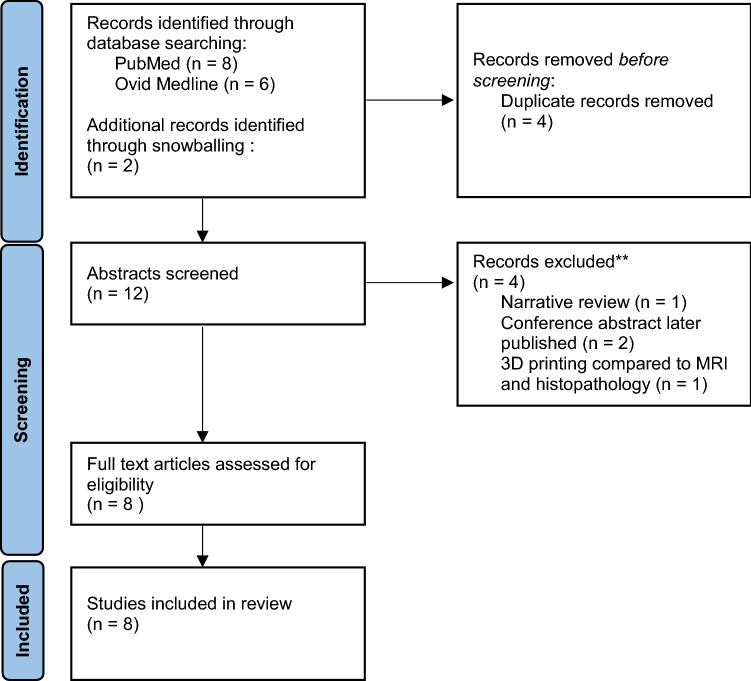
Search strategy as per PRISMA guidelines

Two authors (J.CB. and S.O.) assessed each article for risk bias. Given all articles were non-randomised trials, the ROBINS-I tool was used [19]. Supplementary Table 1 shows the bias score given to each article (see Fig. 2).

## Results

Eight articles were identified for inclusion. Of these articles, five publications (62.5%) reported on the utility of 3D printed models for NS-RARP planning. Two publications (25%) utilised 3D printed prostate models for robotic console simulation, and two publications (25%) used the models as a tool to engage patients. There were five prospective single centre studies, one case series, one technical report and one letter to the editor.

**Fig. 3:**
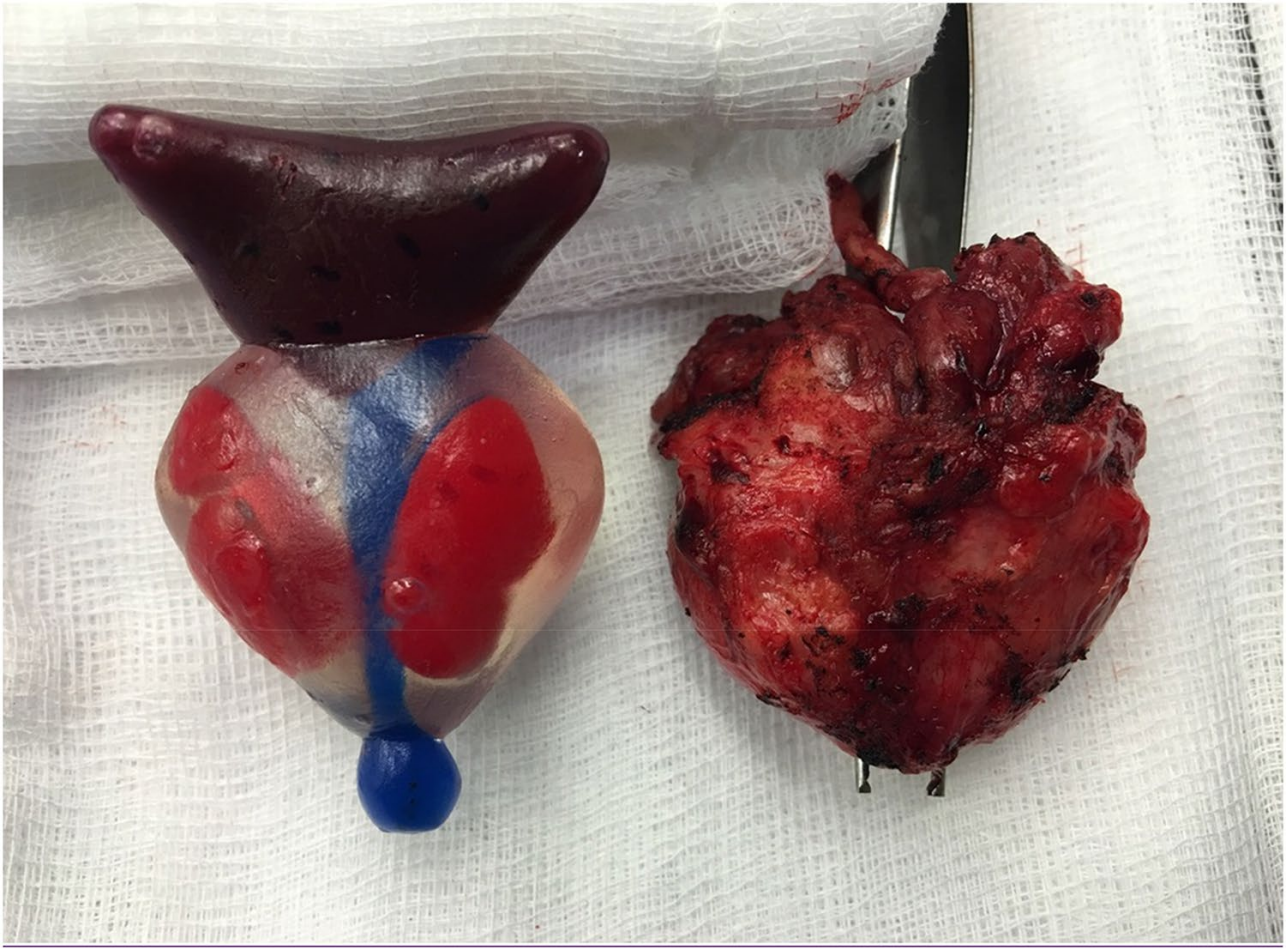
Image of an example 3D printed prostate model (left) with prostate specimen (right). Courtesy of Professor Prokar Dasgupta

A summary of included publications is contained within Table 1. Due to the sparsity of articles concerning the intersection of these two new technologies, all included studies are discussed below (see Fig. 3).

**Table 1:** A summary of included publications

Source	Application	Study type	Study size	Software	Anatomy demonstrated	3D printing technique	Model cost (USD$)
Shin et al. [9]	Presurgical planning	Case series	5 patients (4 T3 and one T2 disease)	Developed by the authors	Prostate, index cancer, neurovascular bundle	Externally printed (Fasotec, Chiba, Japan)	$500
Jomoto et al. [7]	Presurgical planning	Technical report	6 patients (all T2 disease)	ZIOsoft workstation (Tokyo, Japan)	Prostate, seminal vesicles, periprostatic vessels, neurovascular bundles	MF 2000 (Mutoh, Osaka, Japan)	Not disclosed
Chandak et al. [5]	Presurgical planning	Letter	10 patients (all T3 disease)	Mimics (Materialise, Leuven, Belgium)	Prostate, index cancer	Objet500 (Stratasys, Eden Prairie, MN, USA)	$346
Darr et al. [6]	Presurgical Planning	Prospective single center study	10 patients (both RARP and retropubic techniques, 7 T3 and 3 T2 disease)	3D Slicer (Harvard, Boston, MA)	Prostate, seminal vesicles, index cancer	Anycubic Photon (Shenzen, China)	Not disclosed
Porpiglia et al. [8]	Presurgical planning and patient education	Prospective observational study	8 patients (staging not disclosed) and 144 surgeons (both RARP and nephron-sparing surgery)	M3DICS (Torino, Italy)	Prostatic glands, index cancer, neurovascular bundle	Polyjet technology, printer not disclosed	Not disclosed
Wake et al. [12]	Patient education	Prospective observational study	55 patients (PI-RADS X-5, prostate and kidney cancer combined)	Mimics (Materialise, Leuven, Belgium)	Prostate, index cancer, rectal wall, urethra, bladder neck, neurovascular bundles	J750 (Stratasys, Eden Prairie, MN, USA)	Not disclosed
Johnson et al. [10]	Trainee education	Prospective observational study	20 surgeons	TinkerCAD (Autodesk, San Rafael, CA, USA)	Bladder neck and urethra, pelvis	Makerbot Replicator 2 (Makerbot, Brooklyn, NY) used to create silicone moulds	$20 for 3D printed mould, $2.50 of silicone
Witthaus et al. [11]	Trainee education	Prospective observational study	14 surgeons	Mimics (Materialise, Leuven, Belgium)	Prostate, index cancer, seminal vesicles, pelvis, bladder, urethra	Fusion3 F400-S (Fusion3 Design, Greensboro, NC, USA) for 3D printed mould	$75.07 in materials, $160 in personnel hours

## Discussion

### Presurgical planning

Adequate visualisation of the index tumour and the neurovascular bundles are critical to minimising the impacts of NS-RARP on patient quality of life. Shin et al. first described combining the technologies of 3D printing and NS-RARP in 2016 [9]. Using prototype software developed by the group, five patient-specific models comprising the index cancer, prostate and neurovascular bundle were 3D printed. Four patients with T3 disease and one with T2 disease were enrolled in the study. Shin et al. reported that the 3D printed index lesions were useful in planning a wider dissection in the periprostatic tissue surrounding the index lesion to achieve negative surgical margins with partial preservation of the neurovascular bundles. This was validated by concordance between the 3D printed model and histopathology of margins.

Darr et al. confirmed the accuracy of 3D printed patient-specific prostate models derived from MRI when compared to histopathology of the resected specimens via a robot-assisted or retropubic approach [6]. Histopathology proved negative surgical margins in 7 of the 10 patients in this single surgeon study. The three patients with positive surgical margins correlated with those with extracapsular extension on MRI and the 3D printed models. Surgical clearance was achieved in 2 out of 3 of these cases, owing to infiltration of the urethral sphincter in the remaining case making further resection impossible. Specimen dimensions showed a significant correlation between the 3D printed model and histology with regard to length (*p* = 0.01), width (*p* = 0.005), tumour volume (*p* = 0.045), and prostate volume (*p* = 0.002). However, large but not smaller tumours were susceptible to underestimation of up to 30% volume on MRI and subsequent 3D printed models when compared to pathology specimens, suggesting the utility of multiparametric MRI lies in predicting the presence of extracapsular extension, rather than the volume of cancer [6, 20]. In contrast, the prostate volume was over-estimated on MRI when compared with histopathology.

Jomoto et al. placed emphasis on the visualisation of the accessory pudendal arteries (APAs) in addition to the neurovascular bundles in their technical report comprising 6 patients. It has been established that preservation of the neurovascular bundles has been proven to improve postoperative erectile function and continence [16, 17]. In comparison, there has been less emphasis placed on the visualisation of APAs. It has similarly been postulated that preservation of APAs improves postsurgical functional outcomes [21].

Chandak et al. report on a prospective pilot study of 10 patients with localised T3 prostate cancer undergoing NS-RARP [5], based on the IDEAL recommendations [14]. 3D printed prostate models were developed from multiparametric MRI to enhance visualisation. The study reported that tactile interrogation of the model guided decision-making to ensure adequate margins and if nerve-sparing was feasible. Continence was achieved in all ten patients by 12 months. Regarding erectile function, four achieved sufficient erections for intercourse at 24 months, including two patients with anterior tumours who had bilateral nerve sparing. The group reports that larger multicentre randomised control trial is currently underway, with positive margins as the primary outcome. The 3D printed models’ utility as a patient education tool was also informally assessed; however, no objective comparisons were made to standard care without a 3D model.

Porpiglia et al. examined the utility of 3D printed models in presurgical planning and education in 10 patients undergoing robotic-assisted radical partial nephrectomy and 8 patients undergoing NS-RARP [8]. The patients underwent live surgery as part of a Urology conference and patients and surgeons were requested to complete a 10-point Likert scale-based questionnaire on their experience with the 3D printed models.

The 144 respondents were stratified into three groups according to years of experience into expert Urologist (47), Urologist (39), and resident (58) groups. The 3D printed prostate and renal cancer models were rated collectively, with two specific questions investigating the planned surgical approach in each type of cancer. The 3D printed models scored highly in comparison to visualisation of the 3D models on a computer screen (8/10, IQ 9–10), in patient counselling (9/10, IQR 8–9), and in visualising complex anatomy (8/10, IQR 6–8) for presurgical planning (8/10, IQR 6–8). The potential of the 3D printed models as an educational tool also rated highly (9/10, IQR 8–9). Of note was that the utility of the prostate cancer models for the planning of NS-RARP and apex dissection were rated 10/10 (IQR 8–10) and 10/10 (IQR 9–10). There were no statistically significant differences in the responses after stratification by experience of the Urologists (p > 0.05).

Additionally, 18 patients were recruited in the study, of whom 8 underwent NS-RARP. The 3D printed models increased patient satisfaction in their interactions with their surgeons (9/10, IWR 7–9). The models also resulted in a self-rated improvement in their understanding of the disease (10/10, IQR 9–10) and the planned procedure (10/10, IQR 9–10); however, their improved understanding was not objectively assessed.

### Patient education

As discussed above, Porpiglia et al. examined the utility of 3D printed prostate cancer models in their interactions with 8 patients [8]. Compared to this, Wake et al. reported on a patient cohort consisting of 104 prostate cancer patients who underwent RARP [12] as part of their overarching study in which 55 patient-specific anatomical models were collectively 3D printed for patients with renal or prostate cancer. Despite that questionnaire results for the renal and prostate cancer models were not reported separately by Wake et al., this constitutes the largest study to date performed in the field of 3D printing for patient education in any medical discipline, with a total of 200 patients engaged. Despite the differences in cohort sizes, Porpiglia et al. and Wake et al. were in accordance that patient-specific 3D printed models were of significant utility in the therapeutic relationship [8, 12].

Wake et al. also compared patients’ responses to 3D printed models to traditional MRI imaging, augmented reality (AR) or two-dimensional models displayed on a screen [12]. Prostate models were 3D printed on a top-of-the-line J750 machine (Stratasys, Eden Prairie, MN, USA), visualised using the Microsoft HoloLens AR device (Redmond, WA, USA), or shown to the patient on a 2D computer screen. The cost per model of the workflow implemented was not disclosed. Patients were requested to complete a 5-point Likert survey preoperatively to self-evaluate their understanding of their cancer and treatment plan. The three patient groups who received a preoperative model in addition to MRI imaging were asked to complete the survey twice, before and after viewing the 3D model.

The 3D printed models outperformed the other 3D visualisation modalities in all questions. Patients found their anatomy easier to visualise using physical models (9.21 ± 1.49) compared to AR models (7.92 ± 2.84, *p* = 0.04) and more valuable as an aid to understand their disease when compared to AR models (9.11 ± 1.86 vs 8.59 ± 2.05, *p* < 0.05). Surprisingly, patients rated the models viewed using AR as on par across all domains when compared those viewed on a computer screen.

Wake et al. reported that the prostate models were particularly useful in explaining the concept of extracapsular extension when the neurovascular bundle was unable to be preserved. The study’s questionnaire results reported responses as self-evaluated by patients. A next step would have been to objectively assess patients’ understanding of the anatomy of their cancer and their treatment plan before and after viewing the 3D printed models to validate patient self-assessment. Nevertheless, the psychological impact on men undergoing prostatectomy is well known, with lack of preoperative understanding and preparation contributing to significant psychological morbidity postoperatively [22]. Hence, the importance of any preoperative intervention that can lessen anxiety and improve patient preparedness for RARP cannot be understated.

### Simulation and training

There is a need for realistic prostatectomy training simulators for trainees to gain console experience for NS-RARP. In prototyping low cost, accessible simulators, studies have resorted to ballistic gel, fruit, and even poultry models [23, 24].

In keeping with low-cost simulators, Johnson et al. developed a urethrovesical anastomosis trainer costing $2.50 in silicone [10], with the plastic mould printed using inexpensive fused deposition modelling (FDM) 3D printing technology. The model was designed using TinkerCAD (Autodesk, San Rafael, CA, USA) a simple web-based 3D modelling software.

Twenty participants were stratified into three groups based on previous robotic console experience, namely expert, intermediate and novice. The participants’ performance was anonymously recorded for assessment by three reviewers using the previously validated Global Assessment of Robotic Skills (GEARS) [25] and Prostatectomy Assessment and Competency Evaluation (PACE) [26] scores. In addition, the anastomosis performed was rated on a 14-point scale by two Urology fellows and allocated a percentage score for task completion.

A post-task survey using a 5-point Likert scale was used to evaluate the acceptability, face validity, and content validity of the trainer developed. Despite the favourable scores for content (4.15 ± 0.23) and construct validity, face validity was rated lower by participants (3.49 ± 0.23) due to the idealised design of the simulator.

Regarding construct validity, the time taken to complete the exercise decreased with experience. Similarly, completion scores increased with number of years of experience on the robot console. More experienced participants consistently performed higher on the GEARS and PACE scores. Despite the less realistic appearance of the bladder anastomosis trainer, its performance on key simulation metrics, particularly construct validity [27, 28] was consistent with increasing expertise.

Similar to Johnson et al., Witthaus et al. reverse cast inexpensive hydrogels in 3D printed moulds to create a NS-RARP trainer [11]. Both studies developed a simulation tool for urethrovesical anastomosis. However, unlike Johnson et al., the study produced an end-to-end simulator, also incorporating bladder neck dissection, seminal vesicle mobilisation, neurovascular bundle dissection and incorporated a measure for estimated blood loss. The simulator included analogue sensors allowing tension on the neurovascular bundle during dissection to be quantified. The study also simulated a leak test with a multilayered fabricated bladder, and positive surgical margins were simulated with luminol (Science Company, Lakewood, CO, USA) chemiluminescent dye.

Rather than the idealised, task-specific trainer produced by Johnson et al. using simple modelling software [10], the Witthaus et al. trainer was developed from patient MRI data, similar to studies utilising 3D printing technology for preoperative planning of NS-RARP. The simulators were labour intensive to create, with the prostate, seminal vesicles, neurovascular bundles, bladder and urethra sequentially cast from the 3D printed moulds. This was then housed within a 3D printed male pelvis with pelvic floor muscles and fat also cast using hydrogels.

Two blinded surgeons assessed the anonymised video recordings of the task, using the GEARS and Robotic Anastomosis Competency Evaluation (RACE) [29] scores. The 14 participants were stratified, this time into two groups by caseload, into expert and novice groups. This end-to-end simulator developed by Witthaus et al. cost $75.07 in materials and $160 in personnel hours.

With regard to construct validity, novices applied 5 times more maximal pressure and 2.5 times higher average forces when compared to experts. All experts produced a watertight anastomosis on leak test, whereas 6 out of 9 novices had leakage of the simulated urethrovesical anastomosis (p = 0.019). Similarly, none of the experts had positive surgical margins, compared to 7 out of 9 of trainees (*p* = 0.011) and experts accrued significantly less estimated blood loss (230 ml ± 175 ml) when compared to novices (464 ± 186 ml, *p* = 0.071), validating the performance of the model as a simulation tool.

The time to successfully complete bladder anastomosis was 29.5 ± 10.2 min for novices, compared to 11.1 ± 2.6 min for experts. Similarly, Johnson et al. reported that experts performed the fastest anastomosis at 8.7 ± 1.3 min, followed by those with intermediate experience (11.5 ± 0.7 min). As expected, novices took 12 min or longer than this maximum allocated time to complete the task.

Witthaus et al. chose to stratify participants into two groups based on case load, while Johnson et al. divided participants into three groups based on number of years of experience. Despite this, it is evident in both studies that the simulators performed as expected for a valid NS-RARP simulator that was fit for purpose. Participants were rated using RACE scores by Witthaus et al. compared to PACE scoring by Johnson et al., again making direct comparison difficult. However, both groups utilised GEARS scoring out of 30, which confirmed that GEARS score increased with robot exposure; Johnson et al. (experts 25.8 ± 3.1, intermediate 22.4 ± 4.5, novices 10.1 ± 2.7) [10] when compared with Witthaus et al. (experts 29.25 ± 0.96, novices 12.44 ± 3.24) [11]. In both studies, anatomical models made from inert synthetic materials were utilised, negating the need for cadavers or animal models which incur biohazard risks and the expense of a dedicated simulator robot.

### Implementation into practice

Five out of eight (62.5%) of the studies used commercial software which had received regulatory approval for anatomical 3D modelling. Shin et al. used their own prototype software [9], and Darr et al. utilised 3D Slicer (Harvard, Boston, MA, USA) [6, 30], an open-source software. Johnson et al. utilised proprietary web-based software TinkerCAD (Autodesk, San Rafael, CA, USA) [10] to develop their idealised uterovesical anastomosis model for training purposes.

As 3D printed anatomical models become more frequently adopted and enter the surgical mainstream, the ethical and regulatory considerations in implementing this technology will come to the fore. As with all new technologies in surgery, regulation drives reimbursement and implementation into contemporary surgical practice, with the American Medical Association (AMA) introducing provisional reimbursement codes in collaboration with the FDA [31]. This will prompt further exploration into the use of 3D printed models in NS-RARP and other surgical domains such as visualising urethral length as studies have shown this is an important predictor of return of continence [32].

### Economic impact

﻿As the applications of 3D technologies in uro-oncology continue to mature, cost–benefit analysis is necessary. Although low cost FDM technology has been used to create prostate models by Chen et al. [33] where machines retail from a few hundred dollars upwards [1], Wake et al. utilised polyjet technology to 3D print models [12], using the Stratasys J750 printer which retails for hundreds of thousands of dollars, excluding staffing and maintenance costs.

Only Witthaus et al. disclosed the overall cost of their 3D printed prostate models, accounting for labour costs in addition to material costs [11]. Only four (50%) studies reviewed disclosed the cost of their 3D models, ranging from $23–500. In addition, as regulators play catch-up and mandate regulatory approval for segmentation software used to develop patient-specific 3D models, the cost of commercial software licenses must be added to the cost–benefit analysis. These factors must be considered before widespread implementation of this technology into the surgical system is viable.

Ballard et al. studied the feasibility of 3D printing for Orthopaedic and Maxillofacial Surgery by performing a targeted literature financial analysis incorporating 7 studies investigating 3D printed anatomical models and 25 studies investigating 3D printed guides [34]. Across the studies, 3D printing reduced the average operative time by 23 min, saving $1488 per case. In addition to this, regarding NS-RARP there may be further cost savings in the surgical system specific to the procedure, such as reducing the financial burden of postoperative incontinence, erectile dysfunction, and subsequent psychological distress on health services due to improved visualisation of prostate cancer anatomy. Ballard et al. reported that a minimum of 63 3D printed models need to be produced by a surgical department per year to break even when accounting for the start up and running costs of a 3D printing service [34].

### Study limitations

The nascency of 3D printed models to assist NS-RARP contributed to this systematic review’s largest limitation. Despite a broad search strategy augmented with a snowballing approach, only eight publications were identified for inclusion, of which three comprised case studies and a letter to the editor. In addition, the authors were unable to identify any registered clinical trials on this topic [35].

A further systematic review is merited as this field of study matures and larger scale studies become available, particularly given the limitations inherent in systematic reviews comprising small numbers of low-level evidence. The small number of publications identified in the field of NS-RARP and 3D printing, combined with and variability in application of the technologies for preoperative planning [5–9], patient education [8, 12] and simulation [10, 11] makes direct comparison and statistical analysis difficult. Additional difficulties in the systematic review of new technologies arise from publication bias, the low rate of reproducibility of novel studies due to the perceived unattractiveness of reproducing published work, and inadequate details in the methodology for feasible replication [36, 37].

## Conclusion

Despite the nascency of the field, 3D printed models are emerging as a useful tool in NS-RARP for visualising complex anatomy for the purposes preoperative planning, simulation, and patient engagement. However, appropriate clinical practice guidelines, regulatory and reimbursement considerations, and formal cost–benefit analysis must be investigated prior to mainstream implementation into Urological practice.

## Supplementary Information

Below is the link to the electronic supplementary material.

Supplementary file1 (DOCX 16 kb)

## Data Availability

Data transparency: all data generated or analysed during this study are included in this article.

## References

[1] Coles-Black J, Chao I, Chuen J (2017). Three-dimensional printing in medicine. Med J Aust.

[2] Manning TG (2018). Three dimensional models in uro-oncology: a future built with additive fabrication. World J Urol.

[3] Cacciamani GE (2019). Impact of three-dimensional printing in urology: state of the art and future perspectives. A systematic review by ESUT-YAUWP Group. Eur Urol.

[4] Coles-Black J (2021). Utility of 3D printed abdominal aortic aneurysm phantoms: a systematic review. ANZ J Surg.

[5] Chandak P (2018). Three-dimensional printing in robot-assisted radical prostatectomy—an Idea, development, exploration, assessment, long-term follow-up (IDEAL) Phase 2a study. BJU Int.

[6] Darr C et al (2020) Three-dimensional magnetic resonance imaging-based printed models of prostate anatomy and targeted biopsy-proven index tumor to facilitate patient-tailored radical prostatectomy-a feasibility study. Eur Urol Oncol. (Published ahead of print). 10.1016/j.euo.2020.08.004 32873530

[7] Jomoto W (2018). Development of a three-dimensional surgical navigation system with magnetic resonance angiography and a three-dimensional printer for robot-assisted radical prostatectomy. Cureus.

[8] Porpiglia F (2018). Development and validation of 3D printed virtual models for robot-assisted radical prostatectomy and partial nephrectomy: urologists' and patients' perception. World J Urol.

[9] Shin T, Ukimura O, Gill IS (2016). Three-dimensional printed model of prostate anatomy and targeted biopsy-proven index tumor to facilitate nerve-sparing prostatectomy. Eur Urol.

[10] Johnson BA (2019). Design and validation of a low-cost, high-fidelity model for urethrovesical anastomosis in radical prostatectomy. J Endourol.

[11] Witthaus MW (2020). Incorporation and validation of clinically relevant performance metrics of simulation (CRPMS) into a novel full-immersion simulation platform for nerve-sparing robot-assisted radical prostatectomy (NS-RARP) utilizing three-dimensional printing and hydrogel casting technology. BJU Int.

[12] Wake N (2019). Patient-specific 3D printed and augmented reality kidney and prostate cancer models: impact on patient education. 3D Print Med.

[13] Mottet N (2021). EAU-EANM-ESTRO-ESUR-SIOG guidelines on prostate cancer-2020 Update. Part 1: screening, diagnosis, and local treatment with curative intent. Eur Urol.

[14] McCulloch P (2009). No surgical innovation without evaluation: the IDEAL recommendations. Lancet.

[15] Oosterhoff JHF, Doornberg JN (2020). Artificial intelligence in orthopaedics: false hope or not? A narrative review along the line of Gartner's hype cycle. EFORT Open Rev.

[16] Gontero P, Kirby RS (2005). Nerve-sparing radical retropubic prostatectomy: techniques and clinical considerations. Prostate Cancer Prostatic Dis.

[17] Walsh PC, Lepor H, Eggleston JC (1983). Radical prostatectomy with preservation of sexual function: anatomical and pathological considerations. Prostate.

[18] Liberati A (2009). The PRISMA statement for reporting systematic reviews and meta-analyses of studies that evaluate healthcare interventions: explanation and elaboration. BMJ.

[19] Sterne JA, et al (2016) ROBINS-I: a tool for assessing risk of bias in non-randomised studies of interventions. bmj, Volume 355. p i4919 10.1136/bmj.i4919 PMC5062054 27733354

[20] Baco E (2016). A randomized controlled trial to assess and compare the outcomes of two-core prostate biopsy guided by fused magnetic resonance and transrectal ultrasound images and traditional 12-core systematic biopsy. Eur Urol.

[21] Park BJ (2009). The incidence and anatomy of accessory pudendal arteries as depicted on multidetector-row CT angiography: clinical implications of preoperative evaluation for laparoscopic and robot-assisted radical prostatectomy. Korean J Radiol.

[22] Kong EH, Deatrick JA, Bradway CK (2017). Men's experiences after prostatectomy: a meta-synthesis. Int J Nurs Stud.

[23] Lawrentschuk N, Lindner U, Klotz L (2011). Realistic anatomical prostate models for surgical skills workshops using ballistic gelatin for nerve-sparing radical prostatectomy and fruit for simple prostatectomy. Korean J Urol.

[24] Puliatti S, et al (2021) Development and validation of the metric-based assessment of a robotic vessel dissection, vessel loop positioning, clip applying and bipolar coagulation task on an avian model. J Robot Surg. (Published ahead of print). 10.1007/s11701-021-01293-6 34383208

[25] Aghazadeh MA (2015). External validation of global evaluative assessment of robotic skills (GEARS). Surg Endosc.

[26] Hussein AA (2017). Development and validation of an objective scoring tool for robot-assisted radical prostatectomy: prostatectomy assessment and competency evaluation. J Urol.

[27] Gallagher AG, Ritter EM, Satava RM (2003). Fundamental principles of validation, and reliability: rigorous science for the assessment of surgical education and training. Surg Endosc.

[28] Chen G (2020). Three-dimensional printing as a tool in otolaryngology training: a systematic review. J Laryngol Otol.

[29] Khan H (2018). Use of Robotic Anastomosis Competency Evaluation (RACE) for assessment of surgical competency during urethrovesical anastomosis. Can Urol Assoc J.

[30] Fedorov A (2012). 3D Slicer as an image computing platform for the Quantitative Imaging Network. Magn Reson Imaging.

[31] Kemp S (2020). Ethical and regulatory considerations for surgeons as consumers and creators of three-dimensional printed medical devices. ANZ J Surg.

[32] Mungovan SF (2017). Preoperative membranous urethral length measurement and continence recovery following radical prostatectomy: a systematic review and meta-analysis. Eur Urol.

[33] Chen MY (2020). Multi-colour extrusion fused deposition modelling: a low-cost 3D printing method for anatomical prostate cancer models. Sci Rep.

[34] Ballard DH (2020). Medical 3D printing cost-savings in orthopedic and maxillofacial surgery: cost analysis of operating room time saved with 3D printed anatomic models and surgical guides. Acad Radiol.

[35] Witowski J (2018). From ideas to long-term studies: 3D printing clinical trials review. Int J Comput Assist Radiol Surg.

[36] Mullen PD, Ramírez G (2006). The promise and pitfalls of systematic reviews. Annu Rev Public Health.

[37] Dreber A (2015). Using prediction markets to estimate the reproducibility of scientific research. Proc Natl Acad Sci U S A.

