# Supplementary material for: 3D printed patient-specific prostate cancer models to guide nerve-sparing robot-assisted radical prostatectomy: a systematic review
Source: J Robot Surg. 2022 Mar 29;17(1):1–10. doi: 10.1007/s11701-022-01401-0 (PMC9939493; doi:10.1007/s11701-022-01401-0)
Supplement: Supplementary file 1 — Supplementary file1 (DOCX 16 kb) [file 11701_2022_1401_MOESM1_ESM.docx]

**Supplementary data**

**Supplementary Table 1**. Risk of bias evaluation of non-randomized trials included in this review using ROBINS-I tool.

| Study | Study design | Confounding | Selection | Intervention classification | Deviation from intervention | Missing data | Measurement of outcome | Selection of reported result | Overall risk of bias |
| --- | --- | --- | --- | --- | --- | --- | --- | --- | --- |
| Darr et al [6] | Non-randomized trial | Low | Low | NA | Low | Low | Moderate | Low | Moderate |
| Jomoto et al [7] | Non-randomized trial | Low | Low | NA | Low | Low | Moderate | Low | Moderate |
| Shin et al [9] | Non-randomized trial | Moderate | Low | NA | Low | Low | Low | Low | Moderate |
| Johnson et al [10] | Non-randomized trial | Moderate | Low | NA | Low | Low | Moderate | Low | Moderate |
| Witthaus et al [11] | Non-randomized trial | Moderate | Low | NA | Low | Low | Low | Low | Moderate |
| Chandak 2018 [13] | Non-randomized trial | Moderate | Low | NA | Low | Low | Low | Low | Moderate |
| Porpiglia 2018 [15] | Non-randomized trial | Moderate | Low | NA | Low | Low | Moderate | Low | Moderate |
| Wake 2019 [16] | Non-randomized trial | Moderate | Low | NA | Low | Low | Low | Low | Moderate |
